# Peripapillary Retinal Thickness Maps in the Evaluation of Glaucoma Patients: A Novel Concept

**DOI:** 10.5402/2011/146813

**Published:** 2011-09-07

**Authors:** Kayoung Yi, Mircea Mujat, Wei Sun, B. Hyle Park, Johannes F. de Boer, Teresa C. Chen

**Affiliations:** ^1^Glaucoma Service, Department of Ophthalmology, Massachusetts Eye and Ear Infirmary, Harvard Medical School, 243 Charles Street, Boston, MA 02114, USA; ^2^Department of Ophthalmology, Kangnam Sacred Heart Hospital, College of Medicine, Hallym University, Seoul 150-950, Republic of Korea; ^3^Department of Dermatology, Wellman Center for Photomedicine, Massachusetts General Hospital, Harvard Medical School, Boston, MA 02114, USA; ^4^Biomedical Optics Group, Physical Sciences Inc., Andover, MA 01810-1077, USA; ^5^Department of Physics, Boston University, Boston, MA 02215, USA; ^6^Department of Physics and Astronomy, VU University, 1081 HV Amsterdam, The Netherlands

## Abstract

*Purpose*. To show how peripapillary spectral domain optical coherence tomography (SDOCT) retinal thickness (RT) maps can complement retinal nerve fiber layer (RNFL) thickness maps in the evaluation of glaucoma patients. *Methods*. After a complete eye exam with standard fundus photography and visual field testing, normal and glaucomatous eyes were imaged with an experimental SDOCT system. From SDOCT images, RNFL thickness and RT maps were constructed and then correlated with disc photography and visual field testing. *Results*. Two normal eyes of 2 patients and 5 eyes of 4 glaucoma patients were imaged. Although both RNFL and RT maps correlated well with visual field defects, glaucomatous arcuate defects were sometimes more easily identified in the RT maps. *Conclusions*. To our knowledge, this is the first paper to show that peripapillary SDOCT RT maps may provide important supplemental information to RNFL thickness maps in the evaluation of glaucoma patients.

## 1. Introduction

Although glaucoma is primarily a disease of the ganglion cells, the retinal nerve fiber layer (RNFL) is the most commonly imaged layer of the retina in the evaluation of glaucoma patients. Even before the widespread use of imaging technologies, RNFL evaluation has always been an important part of the clinical examination of glaucoma patients [1, 2]. Unlike the ganglion cell layer or other neurosensory retinal layers which are relatively optically transparent, the RNFL is more readily seen on a standard clinical exam and shows fine striations with ophthalmoscopy or slit lamp biomicroscopy with or without a red-free light source. Glaucomatous RNFL changes with red-free photography can even be visualized as early as 6 years prior to glaucomatous visual field defects [1–3]. 

With ultrahigh acquisition speeds and ultrahigh resolution capabilities [4–6], spectral domain optical coherence tomography (SDOCT) technology can image large areas around the optic nerve head and can potentially provide the most comprehensive quantitative evaluation of the RNFL and retina in glaucoma [5]. In contrast to time domain optical coherence tomography (OCT) which only measures RNFL thickness along a circular scan centered on the optic nerve head, SDOCT can create RNFL thickness maps of large areas around the optic nerve head (e.g., 5 × 5 mm areas) [7]. 

A limitation of OCT RNFL thickness measurements is that RNFL thickness measurements are less reliable when the RNFL is thinned, as occurs with glaucoma [8]. In a study of the reliability of RNFL thickness measurements with time domain OCT (StratusOCT, Carl Zeiss Meditec, Dublin, Calif), the coefficients of variation were higher in glaucomatous patients than in normal patients for all the test parameters [9]. Because of the inherent difficulties in obtaining reliable RNFL thickness maps in glaucoma patients, this study investigated whether supplemental SDOCT peripapillary retinal thickness (RT) maps, which are easier to obtain, can also be correlated with fundus photography and visual field testing and whether RT maps may potentially be useful in the clinical evaluation of normal and glaucoma patients. 

To our knowledge, this is the first publication to suggest the use of peripapillary RT maps in the evaluation of glaucoma patients. After a PubMed search, we are also unaware of any previous publications correlating peripapillary retinal thickness maps with disc photography and visual field testing in glaucoma patients.

## 2. Methods

This research adhered to the tenets of the Declaration of Helsinki. Study protocols were approved by the Massachusetts Eye and Ear Infirmary and Massachusetts General Hospital Institutional Review Boards and were in accordance with the Health Insurance Portability and Accountability Act [10]. All volunteers signed informed consents prior to enrollment in the study. Any eyes with retinal or optic nerve diseases other than glaucoma were excluded. 

All volunteers had a complete eye exam which consisted of best-corrected visual acuity, Goldmann applanation tonometry, slit lamp examination, gonioscopy, and fundus examination by a glaucoma specialist (TC). All volunteers were imaged with fundus photography (Topcon TRC 50IX fundus camera (Topcon, Tokyo, Japan) or Visucam Pro NM (Carl Zeiss Meditec, Dublin, Calif)), had visual field testing using the SITA-standard 24-2 program of the Humphrey visual field analyzer 750i (Carl Zeiss Meditec, Dublin, Calif), and underwent SDOCT imaging. 

Patients were defined as having glaucoma if they had (1) characteristic glaucomatous visual field changes and (2) optic nerve head changes characteristic for glaucoma, as defined below. Optic disc abnormalities included one or more of the following: excavation, notching, focal, or diffuse atrophy of neuroretinal rim area, cup-disc asymmetry between fellow eyes greater than 0.2, or disc hemorrhage. Excavation was defined as undermining of the neuroretinal rim; notching was considered if it involved 2 clock hours; atrophy was defined as neuroretinal rim thinning involving 2 or more clock hours. These eligibility criteria were modeled after the Advanced Glaucoma Intervention Study (AGIS) criteria for open-angle glaucoma (Table  1 from Controlled Clinical Trials 1994; 15:299–325) [11]. Primary open-angle glaucoma, normal-tension glaucoma, pseudoexfoliation glaucoma, and chronic angle closure glaucoma patients were included. Chronic angle-closure glaucoma patients had to have at least half of the angle closed by gonioscopy. 

Physiologic cupping was diagnosed when patients had eye pressures under 22 mmHg, vertical cup-disc ratios greater than 0.4, and normal visual field testing. All normal eyes had normal-appearing optic nerves, had normal visual field testing, had refractive errors of less than ±5 diopters, and were never documented to have intraocular pressures higher than 21 mm Hg. 

**Figure 1:**
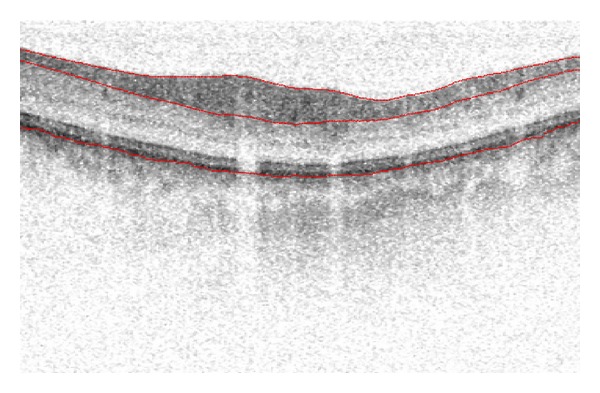
Example of a spectral domain optical coherence tomography (SDOCT) frame that depicts the three boundaries that were automatically determined by our algorithm: (1) top surface of the retina, (2) the posterior retinal nerve fiber layer (RNFL), and (3) the posterior retinal pigment epithelium (RPE). The algorithm sequentially finds the top surface of the retina, the posterior RPE boundary, and then the posterior RNFL boundary. The depth difference between the top surface and the posterior RNFL boundary gives the RNFL thickness while the difference between the top surface and the posterior RPE boundary gives the retinal thickness (RT). The lateral dimension of this frame is 5.81 mm with a scan depth of 1.85 mm. The image was expanded vertically by 2 for better visualization.

The experimental SDOCT instrument was developed at the Massachusetts General Hospital, Wellman Center for Photomedicine. The basic setup has been published previously in detail [7, 12]. For the light source, a superluminescent diode (SLD, Superlum, Russia) with a full width at half maximum (FWHM) spectral width of 50 nm centered at 840 nm was used. The SDOCT data were processed using an algorithm which measured both the RNFL thickness and the RT [7, 12, 13]. The algorithm sequentially finds the top surface of the retina, then the posterior boundary of the retinal pigment epithelium (RPE), and then the posterior boundary of the RNFL. The largest intensity gradient is automatically found at each step of the program. The segmentation algorithm used anisotropic noise filtering and deformable contours to identify continuous boundaries of interest. The depth difference between the top surface and the posterior RNFL boundary gives the RNFL thickness, while the difference between the top surface and the posterior RPE boundary gives the RT. Figure 1 shows an example of a typical SDOCT frame illustrating the three boundaries: the top surface of the retina, the posterior RNFL, and the posterior RPE as estimated by our automatic algorithm. 

Scans of poor quality were excluded from this study. Poor scan quality included either patient inability to complete scanning of the entire optic nerve head region or physician verification of inaccurate automated RNFL or RT boundary determinations due to poor signal strength.

## 3. Results

Seven eyes of 6 patients were enrolled for this study. They were two males and five females with mean age of 61.0 years ±20.7 standard deviations (range 36–83). The demographics and diagnoses of the subjects are listed in Table 1. 

**Figure 2:**
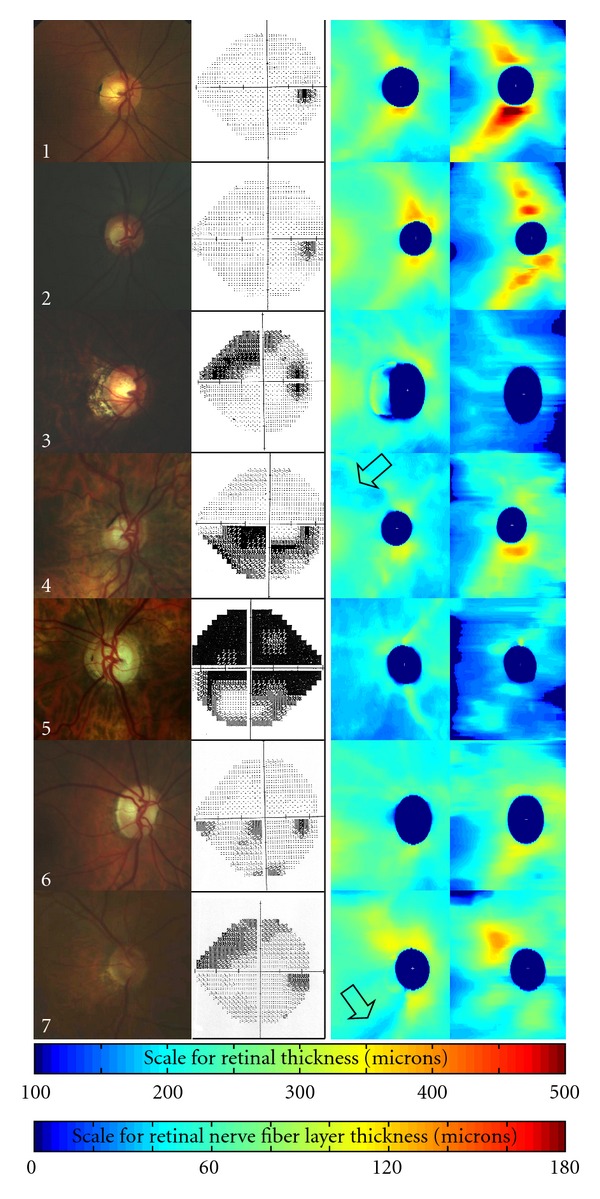
Spectral domain optical coherence tomography (SDOCT) retinal thickness (RT) and retinal nerve fiber layer (RNFL) thickness peripapillary maps in normal (no. 1-2) and glaucoma (no. 3–7) patients. First column: disc photos, second column: visual fields, third column: RT maps, and fourth column: RNFL thickness maps. The thickness scales are seen as the bottom two color bars. The RT map scale ranges from 0 to 500 microns, and the RNFL thickness scale ranges from 0 to 180 microns. Visualization of classic glaucomatous arcuate defects is better seen in RT maps (arrows) than RNFL maps for eyes numbered 4 and 7.

Figure 2 shows the results of the seven eyes. The first column shows the fundus photos. The second column shows visual field testing. The last two columns represent the RT maps and the RNFL thickness maps, respectively. The range of the thickness scale is 0 to 500 microns for the RT maps and 0 to 180 microns for the RNFL thickness maps (Figure 2). 

The first eye is normal and shows maps consistent with known normal anatomy in that the retina and RNFL are both thicker superiorly and inferiorly (Figure 2). Eye number 2 has a larger cup but still has a normal visual field (VF). Both RT and RNFL thickness maps appear normal. Eye number 3 with normal tension glaucoma has a superior nasal step on VF testing. In eye number 3, the RNFL thickness map shows more RNFL thinning inferiorly than superiorly, which also correlates with the VF defect. In the disc photo, thinning of the inferotemporal neuroretinal rim correlates well with both the superior nasal VF defect and the inferotemporal RNFL thinning. The 4th eye shows an inferior arcuate scotoma on VF testing. In this eye, the RT map clearly shows an arcuate area of superior retinal thinning (arrow) which correlates well with the inferior arcuate VF defect. In eye number 4, the arcuate nature of the RNFL defect is not as clearly seen in the RNFL thickness map. In the disc photo, the superotemporal notch with associated superior neuroretinal rim thinning correlates well with the inferior arcuate VF scotoma. Eye number 5 shows a dense superior arcuate with an inferior paracentral defect and an inferior nasal step. The RT map shows a diffusely thinned retina, although with perhaps more thinning inferiorly. The RNFL thickness map also shows diffuse thinning with perhaps more RNFL thinning inferiorly. The 6th eye shows an inferior nasal step and an inferior paracentral scotoma. Both the RT and RNFL thickness maps show greater retinal and RNFL thinning superiorly. Eye number 7 shows vertical cupping with greater thinning of the inferior neuroretinal rim on disc photography, which correlates well with the superior nasal step on VF testing. This superior nasal step also correlates well with the inferior arcuate retinal thinning seen on the RT map (arrow). The RNFL thickness map less clearly shows inferior arcuate RNFL thinning. 

## 4. Discussion

Retinal nerve fiber layer evaluation is a classic part of the evaluation of a glaucoma patient, and imaging devices have been developed to measure RNFL thickness values which can be correlated with visual function [14–17]. RNFL imaging also provides a more objective quantitative evaluation of the RNFL than both the subjective clinical exam and qualitative red-free photography [7]. Imaging devices which calculate RNFL thickness values include the following: scanning laser polarimetry (SLP, GDxVCC, Carl Zeiss Meditec, Dublin, Calif), confocal scanning laser ophthalmoscopy (HRT, Heidelberg Retina Tomograph, Heidelberg Engineering, Heidelberg, Germany), and OCT. SLP however only measures the RNFL thickness indirectly [15, 18–20]. Confocal scanning laser ophthalmoscopy also does not determine true RNFL values, because HRT RNFL thickness values are calculated retinal surface heights from a fixed reference plane 50 microns below the temporal surface of the retina [4]. Of these three imaging technologies, OCT is the only imaging device that measures the RNFL thickness directly [4, 16, 17, 21]. 

Although OCT is the only technology that directly measures the RNFL thickness, accurate peripapillary RNFL thickness measurements in glaucoma patients are often difficult to obtain for a few reasons. OCT RNFL thickness measurements are sometimes less reliable in glaucoma patients [7–9], because decreased RNFL reflectivity, which is associated with glaucomatous damage, makes the posterior RNFL boundary harder to distinguish from the less reflective underlying cellular layers (i.e., the ganglion cell and inner nuclear layers). Especially with the decreased RNFL reflectivity seen in glaucoma, the contrast between the RNFL and the ganglion cell layer/inner plexiform layer (GCL/IPL) is less distinct compared to the contrast between the RPE and surrounding layers. Because of the inherent problems with measuring peripapillary RNFL thickness in glaucoma patients, an alternative measurement of the peripapillary nerve tissue that may be helpful in glaucoma evaluation is RT evaluation. RT measurements have theoretical advantages over RNFL measurements in glaucoma because, unlike the RNFL posterior boundary which becomes less distinct with glaucomatous change, the posterior boundary of the retina (i.e., the highly reflective RPE) is always distinct with even end-stage glaucoma. Therefore, segmentation or identification of this RPE boundary (i.e., posterior retina boundary) is consistently more robust in glaucoma patients, making peripapillary RT determinations potentially more reliable than the peripapillary RNFL thickness measurements. Also, in theory, RT should still have clinical relevance in that thinned RNFL areas should still correspond to areas of thinner RT. Lastly, despite SDOCT's improved resolutions (i.e., about 2 microns for experimental machines) and shorter examination times [5–7, 22–25], SDOCT RNFL thickness measurements are still subject to the inherent measurement variabilities of thinner RNFLs with less reflectivity. In summary, in light of the limitations of peripapillary RNFL thickness measurements in glaucoma patients with both the time domain OCT and SDOCT technologies, peripapillary RT maps may provide more reliable information which is also consistently easier to obtain. 

With OCT imaging of glaucoma patients, RT and RNFL thickness measurements are not usually both used for analyzing the peripapillary region. In glaucoma evaluation, RT measurements have focused on evaluating the macular region of the retina [26–28], and RNFL thickness measurements have usually been used to evaluate the peripapillary retina. Evaluation of RT in the macular region in glaucoma has been used since the macula is the area of the retina where the ganglion cell layer is more than one cell layer thick, and glaucoma has been associated with lower RT values in the macula [27, 28]. SDOCT studies have also shown good correlation of the macular ganglion cell complex with visual field testing [29]. Although SDOCT allows for better 3-dimensional imaging of the macular region [30], macular imaging of the glaucoma patient may ultimately be limited by nonglaucomatous macular pathology such as macular degeneration or diabetes. In these patients with concomitant macular disease, peripapillary RT measurements may be more useful. Another advantage of peripapillary SDOCT RT maps is that it includes the RNFL from the entire retina (100%) compared to macular RT maps which image only about 50% of the ganglion cells of the eye [28]. Therefore, in the current SDOCT study, the peripapillary RT maps include RNFL information from the entire retina (100%) as well as the ganglion cell layer around the optic nerve head. This study proposes that the most comprehensive evaluation of glaucomatous structural changes in one region may be achieved with SDOCT peripapillary RT and RNFL thickness maps (Figure 2) of large areas of the posterior pole (e.g., 6 mm by 6 mm area). 

In this SDOCT study, we correlated structural changes in peripapillary RT and RNFL thickness maps with functional changes in visual field testing. For example, in eyes with glaucoma (Figure 2, eyes numbered 3–7), both RT and RNFL thickness maps showed that areas of superior nerve thinning were associated with areas of inferior visual field loss. Also in Figure 2, areas of inferior RT and RNFL thinning were associated with areas of superior visual field loss. In eyes numbered 4 and 7 (Figure 2), the RT maps more clearly demonstrate typical glaucomatous arcuate defects (Figure 2, arrows) compared to the RNFL thickness maps. These two eyes in particular illustrate how RT maps can supplement and complement RNFL thickness maps. The use of RT maps for glaucoma evaluation also has basis in histology since the total RT includes both the RNFL and the ganglion cell layer, both layers which are affected by glaucoma. Further investigation is necessary to establish a normative database for both RT and RNFL thickness maps. This would enable better determination of glaucomatous changes compared to age-matched normals. 

RT measurements have certain limitations. Because it includes all the retinal layers, any changes of any of these layers by a nonglaucomatous disease process can affect RT maps. For example, diabetic changes or age-related macular degeneration (AMD) can cause significant RT changes in the macular region, which is why our current study focused on RT maps around the optic nerve head. Like all information from other imaging devices or from other subjective diagnostic testing (e.g., visual field testing), RT maps should be considered as supplemental information that ultimately should be correlated with and be consistent with objective clinical exam findings. 

In the patients with glaucoma (eyes numbered 3 through 7, Figure 2), the disc photos show that areas of neuroretinal rim thinning correlate well with both VF testing and SDOCT RT/RNFL thickness maps. Therefore, this study suggests that there is good correlation between structure (i.e., optic nerve head photos, RT/RNFL thickness maps) and function (i.e., visual field testing). 

## 5. Conclusion

With SDOCT, both peripapillary RT maps and RNFL thickness maps can be obtained and can correlate well with neuroretinal rim thinning and visual field defects in glaucoma patients. Even though this is a small case series, it shows the novel concept of peripapillary RT maps that may be another useful parameter for evaluating glaucoma patients, especially when RNFL thickness maps are difficult to interpret or when RNFL thickness maps may be difficult to obtain due to glaucomatous RNFL changes. The use of RT maps however is not meant to substitute for RNFL thickness maps and also should be used with caution in the presence of concomitant diseases that affect retinal layers deep to the RNFL. Structure-function correlations between clinical exam findings, quantitative SDOCT measurements, and visual field testing need further investigation.

## Figures and Tables

**Table 1:** Demographics and diagnoses of the 7 eyes of 6 patients who had spectral domain optical coherence tomography imaging of the peripapillary retina and peripapillary retinal nerve fiber layer.

Eye number	Gender/age (years)	Eye	Diagnosis
1	F/52	OD	Normal
2	M/41	OD	Physiologic cupping
3	M/36	OD	Normal-tension glaucoma
4	F/81	OD	Open-angle glaucoma
5	F/81	OS	Open-angle glaucoma
6	F/73	OD	Pseudoexfoliation glaucoma
7	F/83	OD	Open-angle glaucoma

M: male, F: female, OD: right eye, and OS: left eye.

## References

[1] Tuulonen A, Airaksinen PJ (1991). Initial glaucomatous optic disk and retinal nerve fiber layer abnormalities and their progression. *American Journal of Ophthalmology*.

[2] Sommer A, Katz J, Quigley HA (1991). Clinically detectable nerve fiber atrophy precedes the onset of glaucomatous field loss. *Archives of Ophthalmology*.

[3] Zangwill LM, Medeiros FA, Bowd C, Weinreb RN, Green F, Stamper R (2004). Optic nerve imaging: recent advances. *Essentials in Ophthalmology: Glaucoma*.

[4] Yi K, deBoer JF, Chen TC (2007). Optic nerve head and retinal nerve fiber layer imaging in glaucoma. *Contemporary Ophthalmology*.

[5] Yi K, Chen TC, deBoer JF (2006). Spectral domain optical coherence tomography. *Technology in Ophthalmology*.

[6] Wojtkowski M, Leitgeb R, Kowalczyk A, Bajraszewski T, Fercher AF (2002). In vivo human retinal imaging by Fourier domain optical coherence tomography. *Journal of Biomedical Optics*.

[7] Mujat M, Chan RC, Cense B (2005). Retinal nerve fiber layer thickness map determined from optical coherence tomography images. *Optics Express*.

[8] Gurses-Ozden R, Ishikawa H, Hoh ST (1999). Increasing sampling density improves reproducibility of optical coherence tomography measurements. *Journal of Glaucoma*.

[9] Budenz DL, Chang RT, Huang X, Knighton RW, Tielsch JM (2005). Reproducibility of retinal nerve fiber thickness measurements using the stratus OCT in normal and glaucomatous eyes. *Investigative Ophthalmology and Visual Science*.

[10] Cense B, Nassif NA, Chen TC (2004). Ultrahigh-resolution high-speed retinal imaging using spectral-domain optical coherence tomography. *Optics Express*.

[11] The AGIS Investigators (1994). The advanced glaucoma intervention study (AGIS): 1. Study design and methods and baseline characteristics of study patients. *Controlled Clinical Trials*.

[12] Nassif NA, Cense B, Park BH (2004). In vivo high-resolution video-rate spectral-domain optical coherence tomography of the human retina and optic nerve. *Optics Express*.

[13] Yi K, Mujat M, Park BH (2009). Spectral domain optical coherence tomography for quantitative evaluation of drusen and associated structural changes in non-neovascular age-related macular degeneration. *British Journal of Ophthalmology*.

[14] Greaney MJ, Hoffman DC, Garway-Heath DF, Nakla M, Coleman AL, Caprioli J (2002). Comparison of optic nerve imaging methods to distinguish normal eyes from those with glaucoma. *Investigative Ophthalmology and Visual Science*.

[15] Zangwill LM, Bowd C, Berry CC (2001). Discriminating between normal and glaucomatous eyes using the Heidelberg retina tomograph, GDx nerve fiber analyzer, and optical coherence tomograph. *Archives of Ophthalmology*.

[16] Hwang JM, Kim TW, Park KH, Kim DM, Kim H (2006). Correlation between topographic profiles of localized retinal nerve fiber layer defects as determined by optical coherence tomography and red-free fundus photography. *Journal of Glaucoma*.

[17] Lalezary M, Medeiros FA, Weinreb RN (2006). Baseline optical coherence tomography predicts the development of glaucomatous change in glaucoma suspects. *American Journal of Ophthalmology*.

[18] Zangwill LM, Weinreb RN, Beiser JA (2005). Baseline topographic optic disc measurements are associated with the development of primary open-angle glaucoma: the confocal scanning laser ophthalmoscopy ancillary study to the ocular hypertension treatment study. *Archives of Ophthalmology*.

[19] Weinreb RN, Shakiba S, Zangwill L (1995). Scanning laser polarimetry to measure the nerve fiber layer of normal and glaucomatous eyes. *American Journal of Ophthalmology*.

[20] Bagga H, Greenfield DS, Knighton RW (2003). Scanning laser polarimetry with variable corneal compensation: identification and correction for corneal birefringence in eyes with macular disease. *Investigative Ophthalmology and Visual Science*.

[21] Huang D, Swanson EA, Lin CP (1991). Optical coherence tomography. *Science*.

[22] Leitgeb R, Hitzenberger CK, Fercher AF (2003). Performance of fourier domain vs. time domain optical coherence tomography. *Optics Express*.

[23] de Boer JF, Cense B, Park BH, Pierce MC, Tearney GJ, Bouma BE (2003). Improved signal-to-noise ratio in spectral-domain compared with time-domain optical coherence tomography. *Optics Letters*.

[24] Nassif N, Cense B, Park BH (2004). In vivo human retinal imaging by ultrahigh-speed spectral domain optical coherence tomography. *Optics Letters*.

[25] Chen TC, Cense B, Pierce MC (2005). Spectral domain optical coherence tomography: ultra-high speed, ultra-high resolution ophthalmic imaging. *Archives of Ophthalmology*.

[26] Paunescu LA, Schuman JS, Price LL (2004). Reproducibility of nerve fiber thickness, macular thickness, and optic nerve head measurements using StratusOCT. *Investigative Ophthalmology and Visual Science*.

[27] Lederer DE, Schuman JS, Hertzmark E (2003). Analysis of macular volume in normal and glaucomatous eyes using optical coherence tomography. *American Journal of Ophthalmology*.

[28] Guedes V, Schuman JS, Hertzmark E (2003). Optical coherence tomography measurement of macular and nerve fiber layer thickness in normal and glaucomatous human eyes. *Ophthalmology*.

[29] Cho JW, Sung KR, Lee S (2010). Relationship between visual field sensitivity and macular ganglion cell complex thickness as measured by spectral-domain optical coherence tomography. *Investigative Ophthalmology and Visual Science*.

[30] Chen TC, Zeng A, Sun W, Mujat M, de Boer JF (2008). Spectral domain optical coherence tomography and glaucoma. *International Ophthalmology Clinics*.

